# Melatonin Improved Anthocyanin Accumulation by Regulating Gene Expressions and Resulted in High Reactive Oxygen Species Scavenging Capacity in Cabbage

**DOI:** 10.3389/fpls.2016.00197

**Published:** 2016-03-23

**Authors:** Na Zhang, Qianqian Sun, Hongfei Li, Xingsheng Li, Yunyun Cao, Haijun Zhang, Shuangtao Li, Lei Zhang, Yan Qi, Shuxin Ren, Bing Zhao, Yang-Dong Guo

**Affiliations:** ^1^College of Horticulture, China Agricultural UniversityBeijing, China; ^2^Shandong Huasheng Agriculture Co., LtdShandong, China; ^3^Beijing Agriculture Technology Extension StationBeijing, China; ^4^School of Agriculture, Virginia State UniversityPetersburg, VA, USA

**Keywords:** anthocyanin, cabbage, gene expression, melatonin, reactive oxygen species

## Abstract

In this work, we found, that exogenous melatonin pretreatment improved anthocyanin accumulation (1- to 2-fold) in cabbage. To verify the relationship with melatonin and anthocyanin, an Arabidopsis mutant, snat, which expresses a defective form of the melatonin biosynthesis enzyme SNAT (Serotonin N-acetyl transferase), was employed. Under cold conditions, the foliage of wild-type Arabidopsis exhibited a deeper red color than the snat mutant. This finding further proved, that exogenous melatonin treatment was able to affect anthocyanin accumulation. To gain a better understanding of how exogenous melatonin upregulates anthocyanin, we measured gene expression in cabbage samples treated with melatonin and untreated controls. We found that the transcript levels of anthocyanin biosynthetic genes were upregulated by melatonin treatment. Moreover, melatonin treatment increased the expression levels of the transcription factors MYB, bHLH, and WD40, which constitute the transcriptional activation complex responsible for coordinative regulation of anthocyanin biosynthetic genes. We found, that free radical generation was downregulated, whereas the osmotic adjustment and antioxidant capacities were upregulated in exogenous melatonin-treated cabbage plants. We concluded, that melatonin increases anthocyanin production and benefits cabbage growth.

## Introduction

The pigmentation of most plants is controlled by the relative concentrations of anthocyanin, chlorophyll, and carotenoid pigments. Anthocyanins are the most important group of water-soluble pigments in plants and contribute to the blue, red, or purple colors of leaves, flowers, or fruits. Anthocyanins belong to a group of plant natural products with antioxidant activity and play important roles in protecting humans from diseases. The consumption of vegetables and fruits with these colors has become popular among health-conscious consumers due to their high levels of anthocyanins.

Anthocyanins belong to a class of flavonoids synthesized via the phenylpropanoid pathway. They are synthesized, along with flavonols, from phenylalanine and malonyl-CoA (Winkel-Shirley, 2002). The biosynthesis of anthocyanin pigments and the gene networks, that regulate synthesis have been extensively studied (Grotewold, 2006; Allan et al., 2008). Two classes of genes are involved in anthocyanin biosynthesis, structural genes encoding the enzymes, that directly participate in the formation of anthocyanins and other flavonoids and regulatory genes that control the transcription of structural genes. The structural genes, including phenylalanine ammonia-lyase (PAL), cinnamic acid 4-hydroxylase (C4H), chalcone synthase (CHS), chalcone isomerase (CHI), flavanone 3-hydroxylase (F3H), flavonoid 3′-hydroxylase (F3′H), dihydroflavonol 4-reductase (DFR), leucoanthocyanidin dioxygenase (LDOX), and UDP -glucose: flavonoid 3-O-glucosyltransferase (UFGT), and glutathione S-transferase (GST), encode the corresponding enzymes responsible for the biochemical reactions of anthocyanin synthesis. The second group consists of transcription factors that regulate the structural gene expression in anthocyanin biosynthesis. These transcription factors principally belong to two classes, MYB and bHLH, and are thought to co-operatively regulate the anthocyanin biosynthetic genes with a WD40 protein (Gonzalez et al., 2008; Feller et al., 2011). The transcription factors may form complexes to regulate the expression of the biosynthetic genes, e.g., MYB-bHLH-WDR (MBW) transcription factor complex (Zhang et al., 2003; Xu et al., 2014). Many MYB transcription factors, such as MYB12, have been shown to coordinately regulate the early flavonoid biosynthetic genes CHS, CHI, and F3H (Mehrtens et al., 2005), MYB111 (Stracke et al., 2007). MYB-recognition elements have been identified in the promoters of three genes in Arabidopsis and are important for light responsiveness (Hartmann et al., 1998, 2005). The MYB transcription factors PAP1 and PAP2 together with three potential bHLH partners [transparent testa8 (TT8), glabra (GL3) and enhanced glabra (EGL3)], and the WD40 protein transparent testa glabra 1 (TTG1) participate in the regulation of the late anthocyanin biosynthesis genes DFR, LDOX, and UFGT (Baudry et al., 2004). Arabidopsis plants, that overexpressed PAP1 or PAP2 showed intense purple pigmentation in many vegetative organs throughout development. A detailed analysis of PAP1 over-expressing plants showed that some anthocyanin biosynthetic genes are highly expressed, and the accumulation of anthocyanins is strongly enhanced (Borevitz et al., 2000; Tohge et al., 2005). MYBL2 expression is controlled by negative regulatory feedback that involves an atypical bHLH BES1, which plays a role in the brassinosteroid (BR)-regulated anthocyanin accumulation pathway (Ye et al., 2012). GL3, EGL3, and TT8 have partially redundant roles in the regulation of anthocyanin accumulation. Specifically, EGL3 appears to play a dominant role, as demonstrated by analyses of single, double, and triple mutants (Zhang et al., 2003). These factors can form a ternary complex, that regulates anthocyanin biosynthesis in Arabidopsis seeds (Baudry et al., 2004). A yeast two hybrid analysis revealed that EGL3, GL3, and TT8 interact with TTG1 as well as PAP1 and PAP2. Furthermore, transient expression assays of the DFR promoter have shown that different combinations of EGL3 and GL3 with PAP1 and PAP2 result in very strong activation (Zimmermann et al., 2004).

Many environmental factors affect the biosynthesis of anthocyanin in plants. The effects of light on anthocyanin synthesis have been extensively studied in many plants. Anthocyanin can be induced by broad wavelengths of light, including the UV, visible, and far-red regions (Chalker-Scott, 1999). By absorbing high-energy quanta, anthocyanic cell vacuoles protect chloroplasts from both the photoinhibitory and photooxidative effects of strong light (Gould, 2004). Foliar anthocyanins also act as sunscreens against potentially damaging UV-B radiation (Gould, 2004). In many plants, the accumulation of anthocyanin is also promoted by low temperatures (Leyva et al., 1995), a phenomenon, that is strikingly apparent in autumn leaves and alpine plants. Osmotic stress, such as saline excess, water deficiency, and flooding stress, can all induce anthocyanin accumulation (Chalker-Scott, 1999; Hughes et al., 2013). These environmental or developmental regulations mostly rely on the coordinated expression of anthocyanin biosynthesis genes. Hormone signals have also been shown to be involved in anthocyanin regulations [e.g., induction by abscisic acid (ABA), jasmonate (JA), or cytokinins, and repression by gibberellic acid (GA), ethylene, or BRs; (Peng et al., 2011; Shi and Xie, 2014; Xu et al., 2015)].

Anthocyanins, other flavonoids, and phenolic acids belong to a group of plant natural products with antioxidant activity and play important roles in plant protection from stress. Active oxygen produced under stress has generally been accepted as a detrimental factor that causes the gradual peroxidation of lipid structures (Baryla et al., 2000), antioxidant enzyme inactivation (Teisseire and Guy, 2000), and oxidative DNA damage (Kasprzak, 2002). Many experiments have proven, that anthocyanins can inhibit the formation of free radicals and reduce the level of reactive oxygen species (ROS). For example, anthocyanins inhibit hydroxyl radical generation by chelating ferrous ions and effectively scavenging the super-oxide and hydrogen peroxide generated by mechanical injury, sudden temperature changes, or exposure to strong light (Gould et al., 2002). H_2_O_2_ rapidly diffuses through membranes, which may allow vacuolar anthocyanins to scavenge ROS (Hatier and Gould, 2009). In addition to their function as scavengers of free radicals, flavonoids, and phenolic acids also act as chelating metals, that generate ROS via the Fenton reaction. The presence of the OH group at the 3-position of the flavonoid skeleton is the main structural feature responsible for chelating metal ions, such as iron, copper, zinc, and aluminum (Kidd et al., 2001; Verdan et al., 2011). UV-B radiation modifies the production of anthocyanins (Mazza et al., 2000; Bassman, 2004). The UV-absorbing characteristics of anthocyanins have long been considered evidence for the role of UV protection. Anthocyanins offer multifaceted, versatile, and effective protection to plants under stress: they are the Swiss army knife of the plant kingdom (Gould, 2004).

Melatonin is endogenously produced in all plant species and has recently been identified as a free radical scavenger (Galano et al., 2011; Reiter et al., 2015). Solid evidence implicates melatonin as a growth promoter and rooting agent (Hernández-Ruiz et al., 2004, 2005; Arnao and Hernández-Ruiz, 2007; Hernández-Ruiz and Arnao, 2008; Sarrou et al., 2014; Zhang N. et al., 2014). In addition to its roles in plant development, melatonin plays an important role in plant stress defense (Arnao and Hernández-Ruiz, 2009, 2013, 2014, 2015; Zhang et al., 2015). In our previous study, we found, that melatonin alleviated drought- and saline-induced germination inhibition (Zhang et al., 2013; Zhang H. J. et al., 2014). We also found, that melatonin played an important role in fruit ripening and quality in tomato (Sun et al., 2015). Serotonin N-acetyltransferase (SNAT) is the penultimate enzyme in the melatonin biosynthesis pathway in plants. snat is a SNAT gene-inactivation mutant line generated by T-DNA insertion. The melatonin level in the snat knockout mutant lines was 50% less than that in wild-type Arabidopsis plants (Lee et al., 2015).

In this study, we examined the biochemical and transcriptional changes responsible for the increased anthocyanin accumulation of cabbage sprouts treated with melatonin. We showed, that melatonin treatment upregulated the expressions of both structural genes in the anthocyanin biosynthesis pathway and their transcription factors. Melatonin application resulted in a significant increase in anthocyanin accumulation in cabbage sprouts, and these samples consequently exhibited downregulated ROS levels and upregulated antioxidant activities and markers of osmotic adjustment.

## Materials and methods

### Plant materials

Seeds of white cabbage and red cabbage were sterilized in 5% sodium hypochlorite solution for 10 min, rinsed in distilled water five times, and air-dried prior to melatonin treatment. Sterilized seeds were hydro primed using distilled water and melatonin solution at concentration of 10, 50, 500, and 1000 μmol /L for 12 h. After air-dried to the initial water content, seeds were spread on the Petri dish with three layer of moisture filter paper (Whatman International Ltd, Maidstone, UK) to germinate. Seedlings were cultured under 14-h-light/10-h-dark cycle (23–25°C/15–18°C day/night) environment-controlled cabinets. The 7-day-old seedlings were collected without colorless root to measure the anthocyanin content. For gene expression experiments, seedlings were first cultured in constant dark conditions for 3 days and then light conditions for another 3 days after germination. The samples for Q-PCR were collected every day. Each treatment consisted of 20 dishes with 100 seedlings per dish. All experiments were conducted in triplicate.

Mutant and wild-type *Arabidopsis thaliana* lines were of the Columbia (Col-0) ecotype. T-DNA insertion SNAT-deficient mutants designated *snat* (SALK_032239) was obtained from the Arabidopsis Biological Resource Center (http://abrc.osu.edu/). *Arabidopsis* plants were grown in 7 × 7 cm plots in a controlled environment growth room at 23°C under a 16 h light/8 h dark photoperiod. After 4 weeks' culturing, *Arabidopsis* plants were transferred to a growth chamber with temperature of 5°C, an d photoperiod of 16 h light/8 h dark. This 5°C treatment remained for 14 days.

### Reagents

All chemicals used in experiments were of analytical grade. Melatonin (N-acetyl-5-methoxytryptamine) was purchased from Sigma-Aldrich (St. Louis, MO, USA). All other reagents were purchased from Sinopharm Chemical Reagent (Beijing Co., Ltd, China).

### Total anthocyanin content

Anthocyanin pigments undergo reversible structural transformations with a change in pH manifested by strikingly different absorbance spectra (Pazmino-Duran et al., 2001). Plant material was ground to powder under liquid nitrogen and then was dissolved in a 0.025 M potassium chloride buffer, pH = 1.0, and 0.4 M sodium acetate buffer, pH = 4.5. The absorbance of each solution was measured at 510 and 700 nm, against a blank cell filled with distilled water.

The absorbance (A) of the diluted sample was calculated by the following formula:

(1) $$\begin{array}{r} \text{A}=\left({\text{A}}_{510}-{\text{A}}_{700}\right){\;}_{\text{pH}1.0}-\left({\text{A}}_{510}-{\text{A}}_{700}\right)\text{ p}{\text{H}}_{4.5} \end{array}$$

The monomeric anthocyanin pigment concentration in the original sample was expressed in equivalence of cyaniding-3-glucoside that is the main anthocyanin in cabbage, and calculated by the following formula:

(2) $$\begin{array}{r} \text{Monomeric anthocyanin pigment}\left(\text{mg}∕\text{L}\right)=\left({\text{A}}^{*}\text{MW}{\;}^{*}1000\right)∕\\ \left(E^{*}1\right) \end{array}$$

Where MW is the molecular weight of cyanidin-3-glucoside in 449.2; and E is the molar absorptivity, which equal to 26, 900 for cyanidin-3-glucoside.

### Evaluation of ROS and antioxidant enzymes

The content of ROS and the activities of antioxidant enzymes were measured following the method described in our previous work (Zhang et al., 2013). The ROS in this research included H_2_O_2_, OH,· and ${\text{O}}_{2}^{-1}$. The antioxidant enzymes in this research included superoxide dismutase, catalase, peroxidase, and ascorbate peroxidase.

### Lipid peroxidation measurements

The level of lipid peroxidation of each sample was measured as malondialdehyde (MDA) content determined by reaction with 2-thiobarbituric acid (TBA) reactive substances as described in our previous paper (Zhang et al., 2013). A 0.5 g leaf sample was homogenized in 5 ml 0.1% TCA. The homogenate was centrifuged at 10,000 g for 5 min. Four milliliters of 20% TCA containing 0.5% TBA were added to 1 ml of the supernatant then incubated in boiling water for 20 min. The reaction was stopped by placing the reaction tubes on ice. MDA absorption was measured spectrophotometrically at 450, 532, and 600 nm. The concentration of lipid peroxides was thus quantized in terms of the MDA concentration and expressed as nmol/g.

### Proline determination

The sprouts (200 mg) were homogenized with 4 ml of 3% sulphosalicylic acid using a mortar and pestle. The homogenate was centrifuged at 5000 × g for 10 min at 4°C. To 1 ml of the supernatant, 1 ml of glacial acetic acid, and 1 ml of acid ninhydrin was added and boiled for 1 h. After cooling 4 ml of toluene was added and shaken for 30 s. After separating the layers, the upper (toluene) was collected and absorbance was measured at 520 nm on a spectrophotometer (UV2800A; UNICO, Shanghai, China). Proline content was determined using a calibration curve prepared with known concentrations. All tests were repeated at least three times, and the results are presented as means ± SD.

### PAL activity assay

Phenylalanine ammonialyase activity was determined according to Flores et al. (2005) with slight modifications. Total protein was extracted from 500 mg (FW) sprouts in 1 ml extraction buffer (50 mM borate buffer, pH 8.8 containing 5 mM β-mercapto ethanol and 2% w/v polyvinyl pyrrolidone from Sigma, USA). The reaction mixture contained 0.25 ml of enzyme extract, 0.75 ml extraction buffer, 0.35 ml double-distilled water, and 0.15 ml 100 mM L-phenylalanine (Sigma). The reaction mixture was incubated for 30 min at 40°C, and terminated by adding 50 μl of 2.5 M HCl. PAL activity was determined by measuring the concentration of E-cinnamic acid produced at A290 (Flores et al., 2005). Protein concentration was determined using Bradford reagent (Bio-Rad, USA). PAL activity was expressed as micromole E-cinnamic acid in 1 min per gram protein.

### Real-time quantitative PCR (qPCR) analysis

Total RNA was extracted from the samples using TRIzol reagent according to the manufacturer's protocol (Invitrogen, Burlington, ON, Canada). The primers of selected genes were designed using Primer-priemer 5 software (PREMIER Biosoft, Palo Alto, CA, USA) and synthesized by Sangon. The primer pairs are summarized in Table S1. The cDNA was synthesized from 1 μg of total RNA using PrimeScript RT reagent Kit (Takara, Da Lian, China) in 20 μl of reaction mixture. The amount of the amplified DNA was monitored by fluorescence at the end of each cycle using 7500 Real-Time PCR System (Applied Biosystems). Each plate was repeated three times in independent runs for all reference and selected genes. Gene expression was evaluated by the 2^−Δ*ΔCt*^ method (Livak and Schmittgen, 2001).

### Melatonin extraction and analysis

Melatonin was extracted from cabbge seeds and seedlings according to the method described in our previous work (Zhang H. J. et al., 2014). Approximately 1 g frozen samples were ground into powder with liquid nitrogen and homogenized with 10 mL methanol. After centrifugation at 11,417 g at 4°C for 15 min, the supernatants were collected and dried using nitrogen gas. The extracts were then dissolved in 5% methanol and purified using a C18 solid phase extraction (SPE) cartridge (Waters, Milford, MA, USA). The cartridge was next washed with 10 mL 5% methanol, and melatonin was eluted finally at a natural flow rate with 2 mL 80% methanol. The extract was subsequently filtered through a 0.22 μm PTFE syringe filter before UHPLC-ESI-MS/ MS analysis. Melatonin determination and quantification was analyzed using a UHPLC-ESI-MS/MS (UHPLC-1290 Series and a 6460 QqQ-MS/MS; Agilent Technologies, Waldbronn, Germany) with an Agilent SB-C18 column (4.6 9 50 mm; 1.8 l m; Agilent Technologies, Santa Clara, CA, USA).

## Results

### Melatonin treatment increased anthocyanin concentration in cabbage seedlings

**Figure 1:**
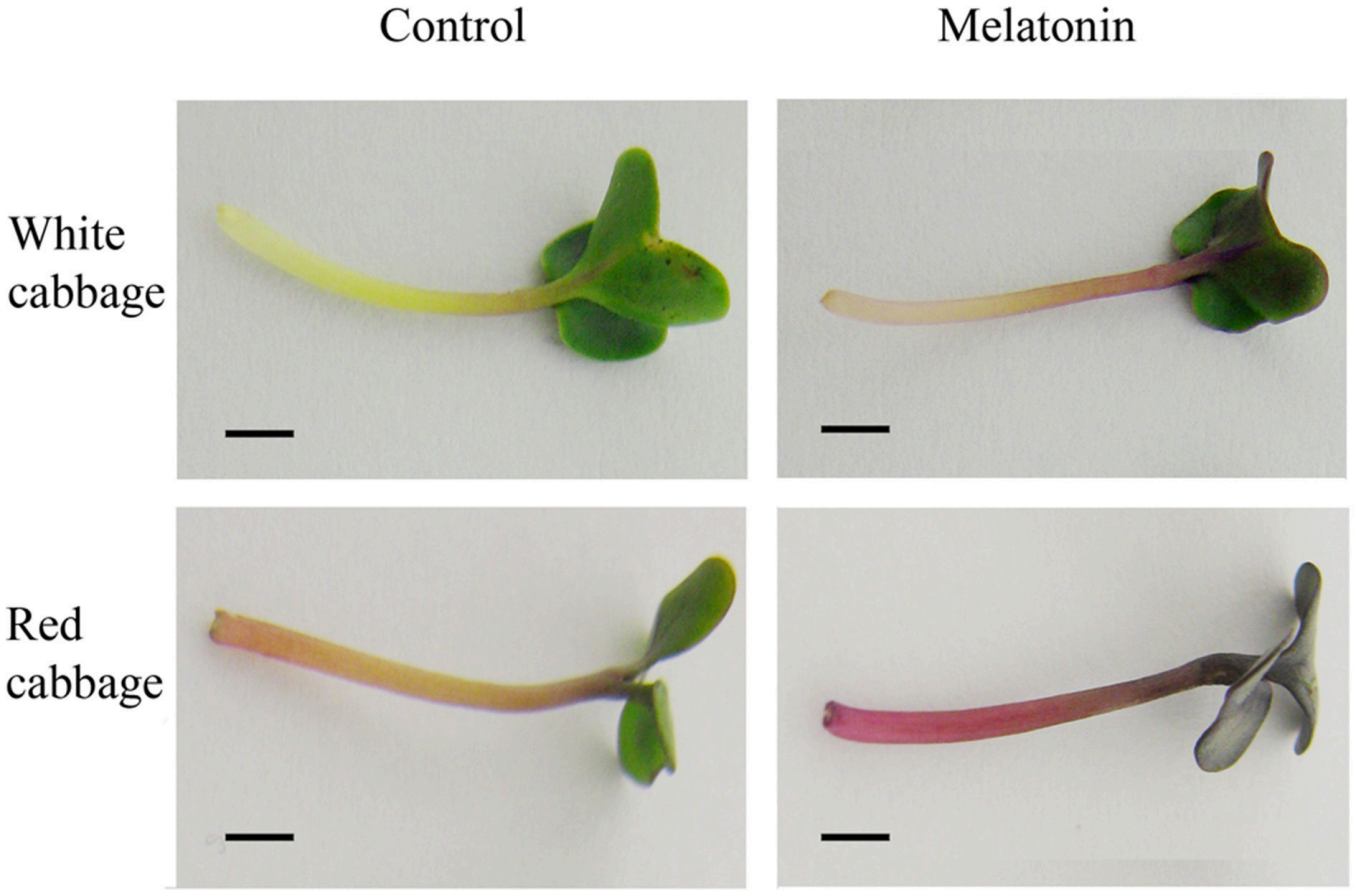
**Melatonin treatment influenced the color depth in white cabbage and red cabbage**. The concentration of melatonin pretreatment is 1000 μmol /L in white cabbage and 100 μmol /L in red cabbage.

**Figure 2:**
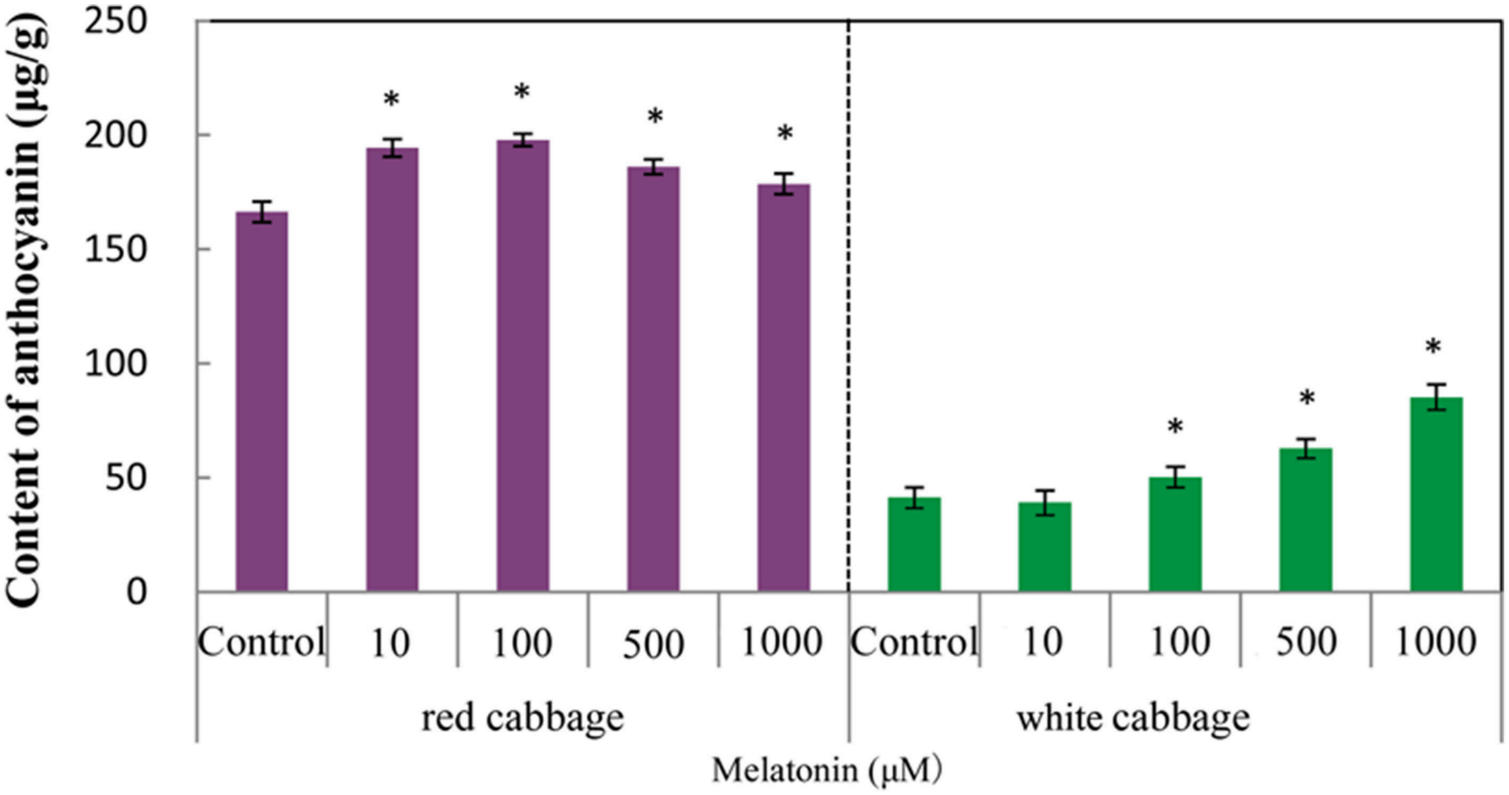
**The anthocyanin levels in white cabbage and red cabbage**. Vertical bars at each column represent standard deviation of three replications. ^*^Significant difference between this column and control at *P* < 0.05.

We analyzed the anthocyanin content in white and red cabbage sprouts pretreated with melatonin and untreated controls. Figure 1 shows, that melatonin-treated cabbage sprouts exhibited darker hypocotyls and cotyledons (Figure 1). The total anthocyanin contents of the two cabbage cultivars are presented in Figure 2. To assess the effect of melatonin on the anthocyanin accumulation in cabbage sprouts, the sprouts were pre-treated with four concentrations of melatonin (10, 100, 500, and 1000 μM/L). Melatonin promotes anthocyanin accumulation in both red and white cabbage. In red cabbage, the four concentrations of melatonin all significantly increased anthocyanin content of sprouts. Specifically, the anthocyanin concentration was highest in red cabbage sprouts treated with 100 μM/L melatonin (198 μg/g FW, 18.7% higher than, that in the control). All concentrations of melatonin pretreatment except 10 μmol/L, which was the lowest concentration, significantly increased the anthocyanin content in white cabbage. The most effective concentration was 1000 μmol/L, which doubled the anthocyanin content in white cabbage compared with the control. The results indicated that melatonin improved the anthocyanin content of cabbage.

### Melatonin-deficient arabidopsis mutant accumulated low levels of anthocyanin

**Figure 3:**
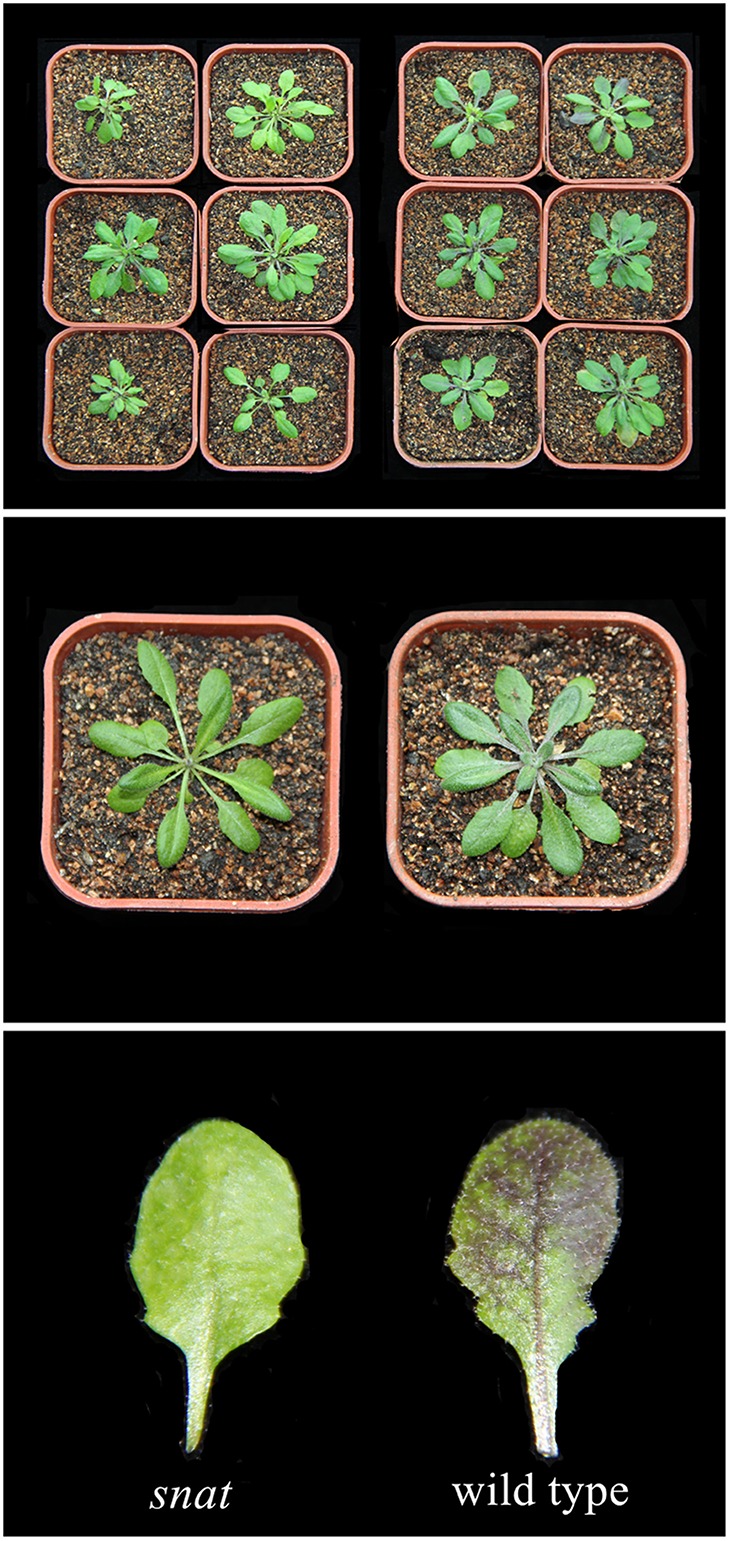
**Anthocyanin accumulation in wild type ***Arabidopsis*** and melatonin defect mutant**.

To further confirm the relationship between melatonin and anthocyanin synthesis, we studied a T-DNA insertion SNAT-deficient mutant that accumulates low levels of melatonin. SNAT is the penultimate enzyme in the melatonin biosynthesis pathway in plants. The melatonin level in the snat knockout mutant lines was 50% less than, that in wild-type Arabidopsis Col-0 plants (Lee et al., 2015). Wild-type and mutant seedlings exhibited color differences after 10 days of cold treatment (Figure 3). The seedlings of the wild-type Columbia ecotype darkened after cold treatment, whereas the melatonin-deficient mutant seedlings were not as dark as the wild-type seedlings. Because the snat mutant accumulates low levels of melatonin and correspondingly accumulates less anthocyanin than the wild type, we speculated that melatonin positively affects anthocyanin accumulation.

### Melatonin treatment upregulated the activity of phenylalanine ammonia-lyase

**Figure 4:**
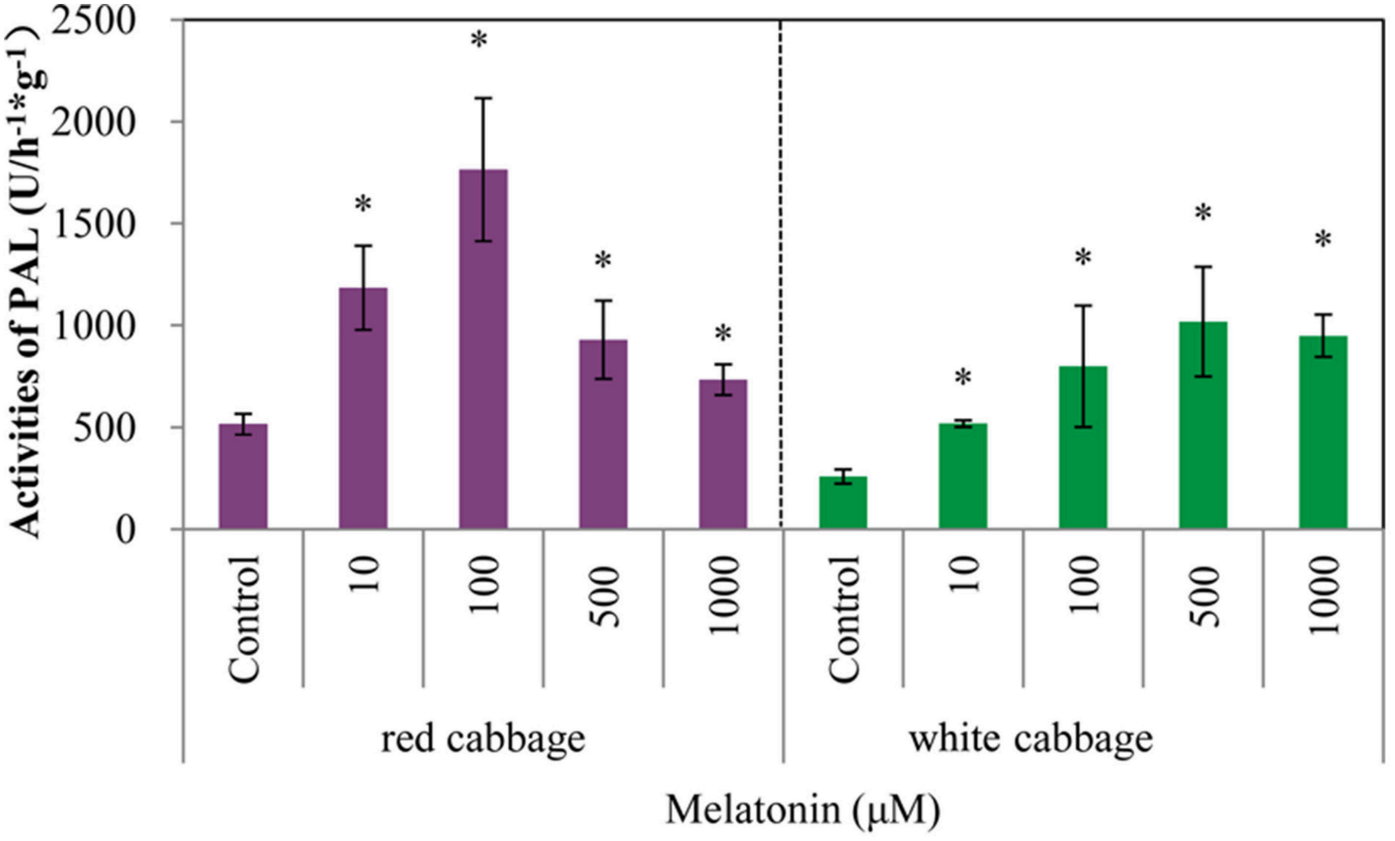
**Melatonin treatment affected PAL activities in white cabbage and red cabbage**. Vertical bars at each column represent standard deviation of three replications. ^*^Significant difference between this column and control at *P* < 0.05.

Phenylalanine ammonia-lyase initiates anthocyanin biosynthesis (Feng et al., 2008). The anthocyanin contents of white cabbage and red cabbage generally correlated with the PAL activities (Figure 4). Melatonin treatment significantly increased the activity of PAL in a concentration-independent manner. The maximum PAL activity in melatonin-treated samples was 4-fold higher than the level of the control. The PAL activity in red cabbage was higher than, that in white cabbage, which is consistent with the anthocyanin biosynthesis in these species. Figures 2, 4 show a positive linear relationship between the PAL activity and the amount of accumulated anthocyanin. Therefore, although PAL participates in the early steps of the synthesis of numerous phenolic and flavonoid compounds, its activity significantly affects anthocyanin synthesis.

### Effect of melatonin treatment on the expression levels of genes along the anthocyanin biosynthetic pathway

To assess whether the increase in the anthocyanin concentration following melatonin treatment is due to an increase in its biosynthesis, the expression levels of transcripts that encode ten anthocyanin biosynthesis genes (*PAL, C4H, CHS, CHI, F3H, F3*′*H, DFR, LDOX, UFGT*, and *GST*) were measured using quantitative real-time PCR.

**Figure 5:**
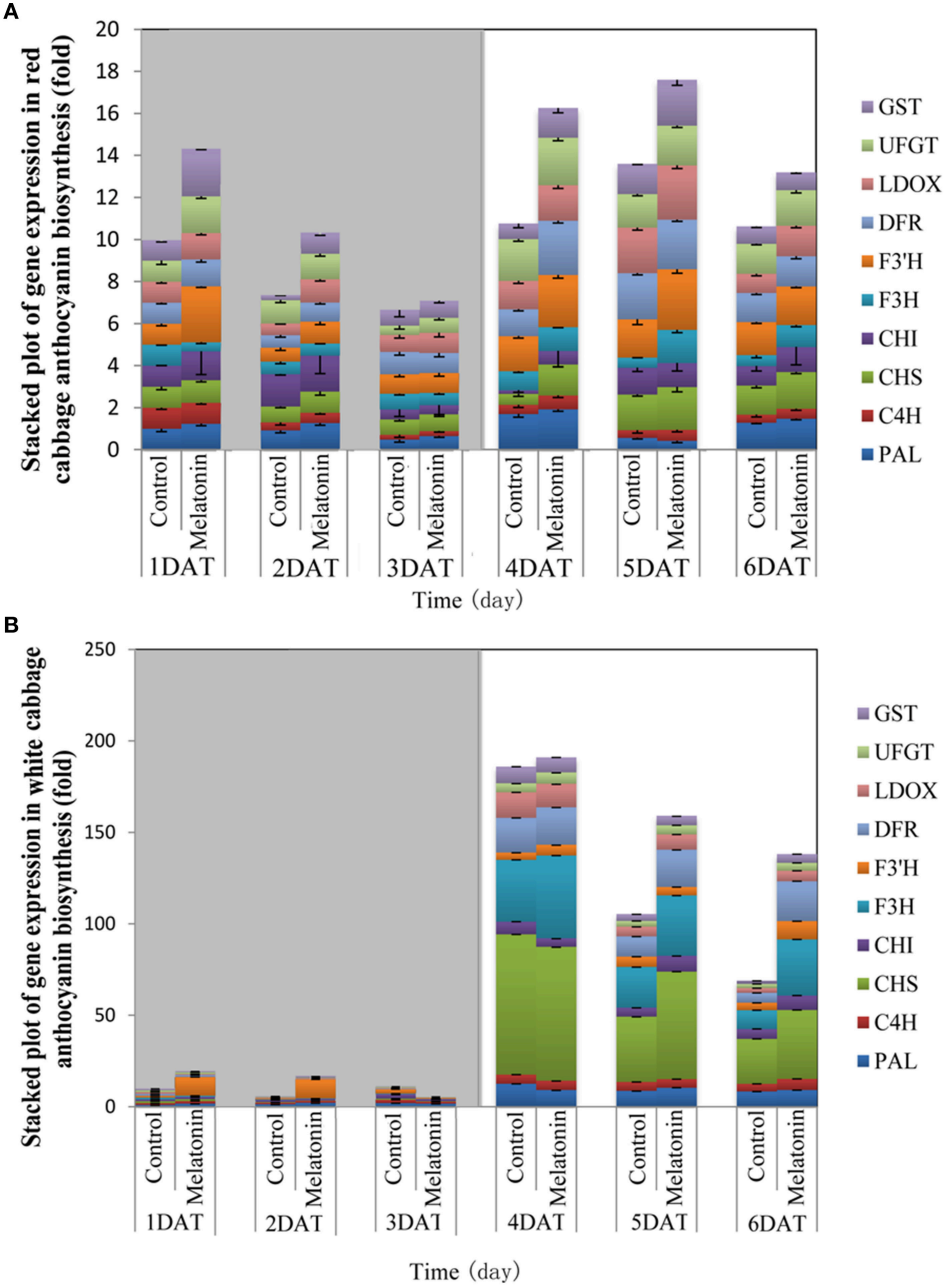
**The expression patterns of anthocyanin synthesis genes in red cabbage (A) and white cabbage (B) in dark and light conditions**. Vertical bars at each column represent standard deviation of three replications.

The expression patterns of the ten biosynthesis genes in white and red cabbage under dark and light conditions are shown in Figure 5. Under dark conditions, the expression levels of anthocyanin biosynthesis genes were low and increased when exposed to light. This phenomenon was more marked in white cabbage than in red cabbage (Figure 5). The expression levels of anthocyanin biosynthesis genes were upregulated by melatonin treatment under both dark and light conditions. The expression levels of these ten genes were low when seedlings were maintained in the dark. Melatonin treatment tended to increase the expression levels of these biosynthesis genes in both white and red cabbage, despite their low expression levels in the dark. When light was added to the culture environment, the expression levels of genes significantly increased. In white cabbage, the expression levels of genes increased 100-fold when stimulated by light (Figure 5B). This change indicated that the expression of anthocyanin biosynthesis genes is trigged by light in white cabbage, whereas these genes maintain a relative high expression in red cabbage under both light and dark conditions.

**Figure 6:**
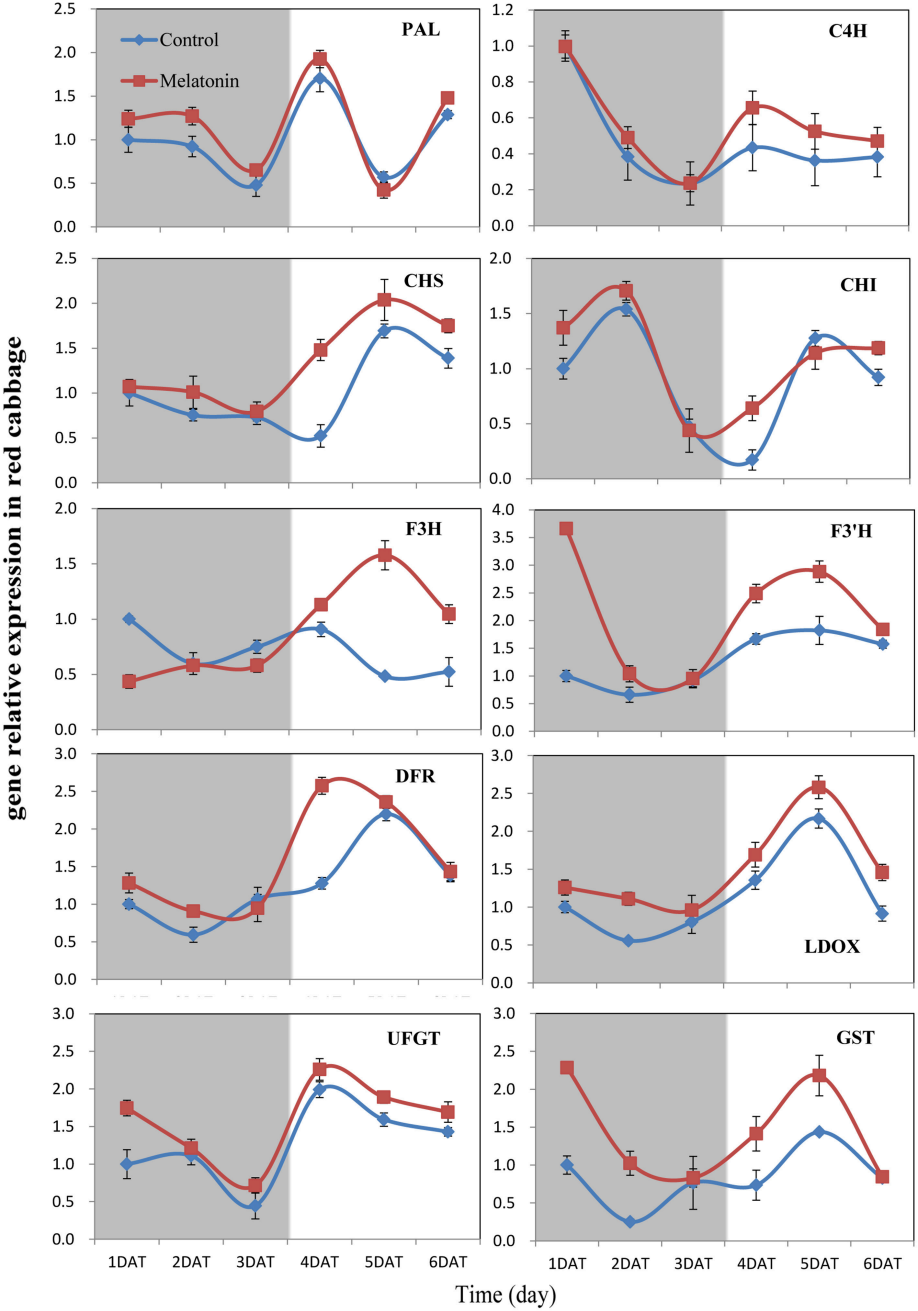
**Expressions of anthocyanin synthesis genes in red cabbage under dark and light conditions**. The concentration of melatonin pretreatment in red cabbage is 100 μmol /L. Vertical bars at each column represent standard deviation of three replications.

**Figure 7:**
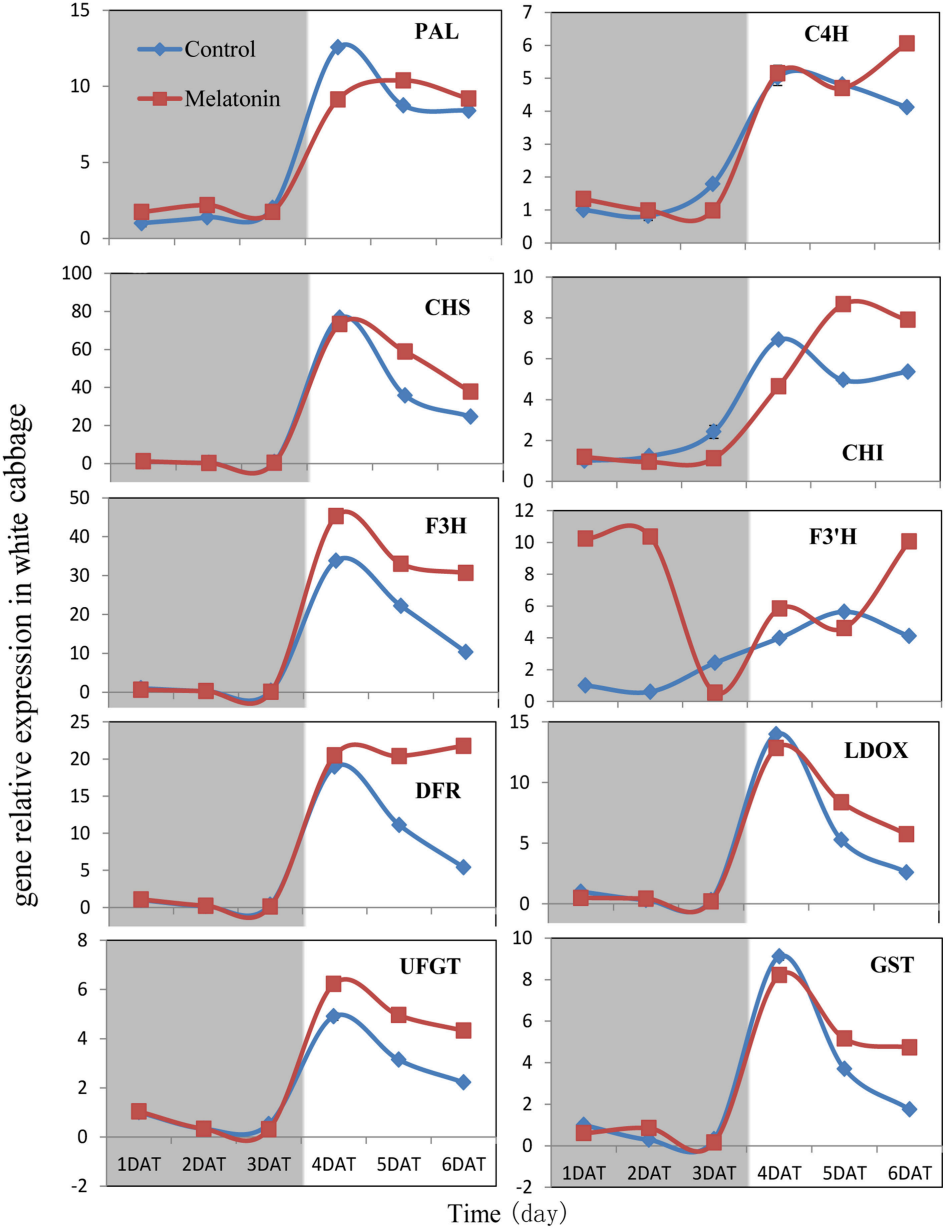
**Expressions of anthocyanin synthesis genes in white cabbage under dark and light conditions**. The concentration of melatonin pretreatment in white cabbage is 1000 μmol /L. Vertical bars at each column represent standard deviation of three replications.

PAL is the first committed enzyme in the anthocyanin biosynthesis pathway, that leads to the production of many flavonoids. This enzyme catalyzes the formation of trans-cinnamic acid from phenylalanine. The melatonin-treated samples expressed higher levels of PAL at four of six tested time points (Figures 6, 7). This finding indicated, that melatonin modulates the expression of genes starting during the early steps in anthocyanin biosynthesis. C4H is a cytochrome P450 monooxygenase that catalyzes the hydroxylation of trans-cinnamic acid at the C-4 position to yield 4-coumaric acid. In the dark, the expression levels of *C4H* of control and melatonin treated samples were similar. When light was introduced to the culture environment, the expression levels of *C4H* in melatonin-treated samples were much higher than that in the control (Figures 6, 7). CHS catalyzes the formation of a triketide intermediate from p-coumaroyl-CoA and three molecules of malonyl-CoA; the subsequent spontaneous cyclization of the triketide intermediate then results in the formation of naringenin chalcone. When exposed to light, melatonin also strongly stimulated the expression of *CHS* in both white and red cabbage (Figures 6, 7). CHI catalyzes the stereospecific cyclization of naringenin chalcone to (2S)-naringenin. The expression of the *CHI* gene differed between white cabbage and red cabbage. In white cabbage, the expression levels of *CHI* were low in the dark in both control and melatonin-treated samples. After light treatment, the expression levels of *CHI* significantly increased. Conversely, the expression levels of *CHI* were initially high in red cabbage, and melatonin-treated samples expressed high levels of *CHI* both under dark and light conditions (Figures 6, 7). This difference may be responsible for the fact, that red cabbage synthesized more anthocyanin than white cabbage. F3H catalyzes the oxygenation at the 3-position of flavanone [(2S)-naringenin] to form dihydroflavonol (dihydrokaempferol), and this reaction concomitantly produces CO_2_ and succinate from oxygen and 2-oxoglutarate as co-substrates. The expression patterns of *F3H* were similar in white and red cabbage: melatonin-treated samples expressed higher levels of *F3H* than control samples (Figures 6, 7). F3′H is a cytochrome P450 monooxygenase committed in the hydroxylation of the 3′-position of the flavonoid B-ring. This enzyme accepts either dihydrokaempferol or kaempferol as a substrate and converts it to dihydroquercetin or quercetin, respectively. Melatonin appeared to particularly promote the expression of *F3*′*H*. Melatonin-treated samples expressed high levels of this gene during initial seedling growth, i.e., 1 day after germination (Figures 6, 7). As the plant continued to grow, the expression levels of this gene tended to be consistent among the two treatment groups. Generally, melatonin significantly upregulated the expression of *F3*′*H*. DFR catalyzes the reduction reaction of dihydroflavonol to leucoanthocyanidin. The expression of *DFR* showed similar response to melatonin with *F3H* (Figures 6, 7). The enzyme LDOX catalyzes the formation of anthocyanidin from leucoanthocyanidin, producing 2-oxoglutarate and oxygen as co-substrates. In red cabbage, the expression of *LDOX* remained higher in melatonin-treated samples than in control samples, both in dark and light conditions (Figure 6). Conversely, this gene only slightly responded to melatonin in white cabbage under light conditions (Figure 7). UFGT catalyzes the glycosylation of flavonoid skeletons and utilizes UDP-sugars as sugar donors. The expression patterns of this gene in white cabbage and red cabbage were similar to those of *LDOX* (Figures 6, 7). Anthocyanins are unstable at pH > 4.0. GST protein is the flavonoid carrier and forms conjugates with anthocyanins, preventing them from oxidizing. It is involved in the transport of anthocyanins from the cytosol to the vacuole. Melatonin-treated samples showed improved expression levels of *GST* compared with control samples, especially in red cabbage (Figure 6). In summary, we found, that melatonin treatment upregulated the expressions of structural genes in the anthocyanin biosynthesis pathway. Furthermore, this finding is consistent with the increase in anthocyanin levels in cabbage in response to melatonin.

### Induction of regulatory gene expression in response to melatonin under dark and light conditions

**Figure 8:**
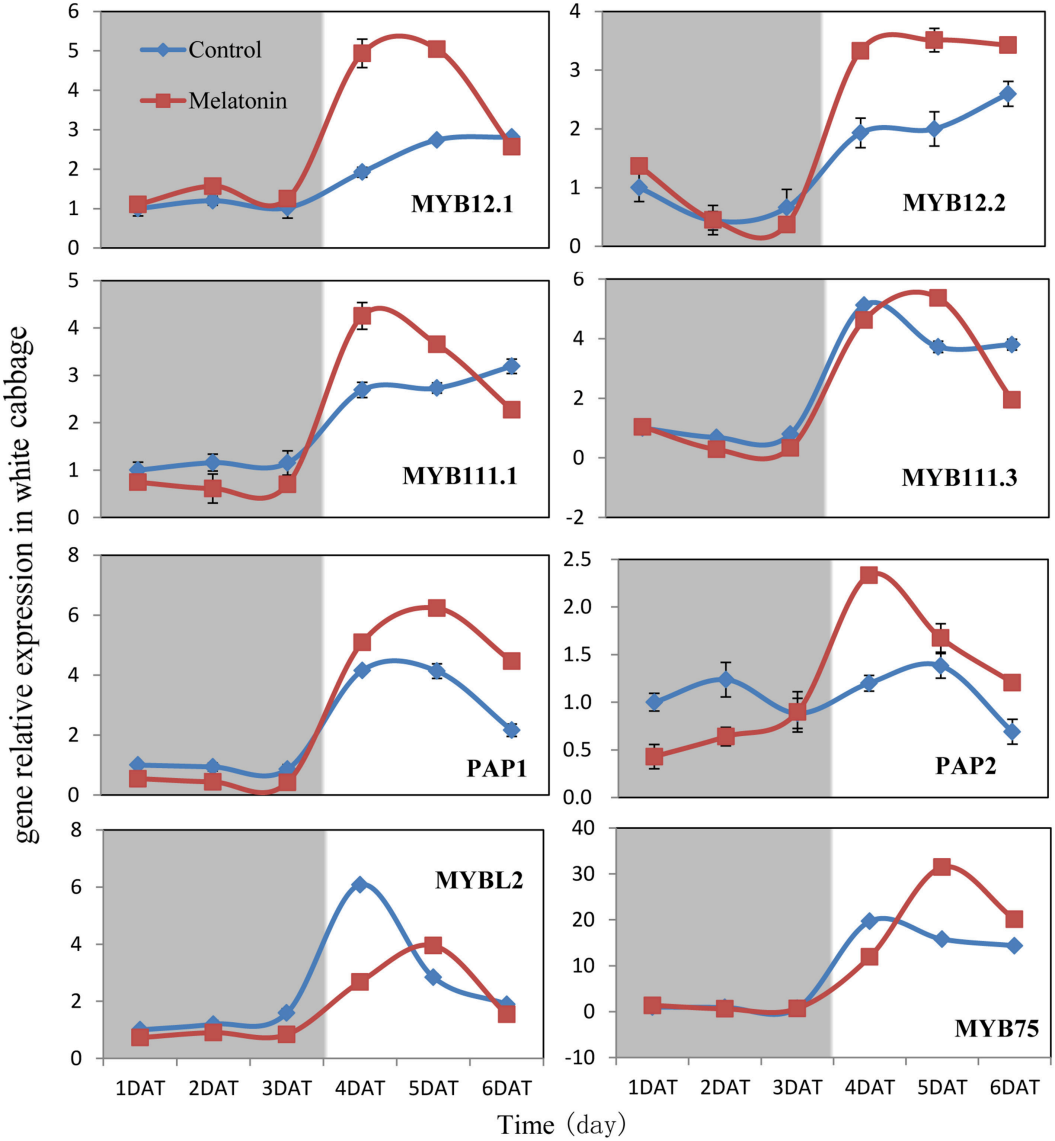
**Expressions of MYB type transcription factors participate in anthocyanin synthesis in white cabbage**. The concentration of melatonin pretreatment is 1000 μmol /L. Vertical bars at each column represent standard deviation of three replications.

**Figure 9:**
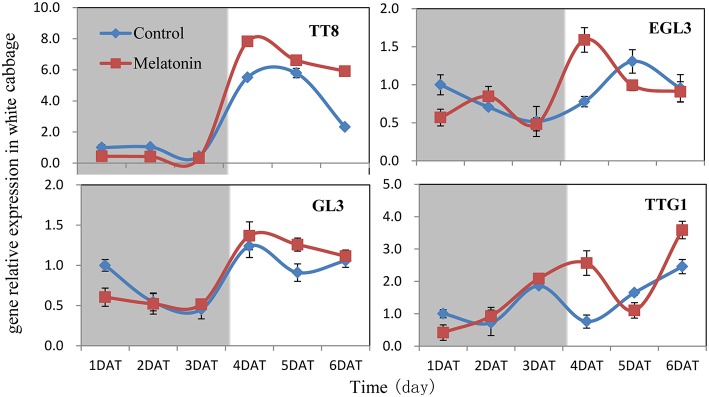
**Expressions of bHLH (***TT8***, ***EGL3***, ***GL3***) and WD40 type (***TTG1***) transcription factors participate in anthocyanin synthesis in white cabbage**. The concentration of melatonin pretreatment is 1000 μmol /L. Vertical bars at each column represent standard deviation of three replications.

We aimed to investigate the accumulation of transcripts corresponding to the anthocyanin regulatory genes in response to melatonin in dark-grown and light-grown seedlings and to examine possible correlations between their expression patterns and those of the structural genes. Transcripts corresponding to the 8 MYB transcription factor genes analyzed were expressed at a very low level in dark-grown samples (Figure 8), and the expression levels of control and melatonin-treated samples did not significantly differ. After light treatment, we observed a strong increase in the transcript levels of all MYB transcription factors. Melatonin-treated samples expressed higher levels than control samples (Figure 8). *MYBL2* is an exception to this pattern because it is a negative regulator of anthocyanin biosynthesis genes. This finding was consistent with changes in the expression of structural genes. Anthocyanin biosynthesis genes are regulated by the interaction of the transcription factors MYB, bHLH, and WD40. The bHLH transcription factors participate in anthocyanin biosynthesis regulation, including *TT8, EGL3*, and *GL3*. TTG1 is a WD40 protein that correlates with anthocyanin biosynthesis. In the dark, the expression levels of these genes were similar between the control and melatonin-treated samples for all points except for the first, at which melatonin-treated samples expressed lower levels than the control. The expression of *TT8* significantly increased after illumination (Figure 9) and peaked to a level that was six-fold higher than, that of the dark-grown plant 1 day after light treatment. Moreover, the maximum expression of *TT8* in melatonin-treated samples was 8-fold higher than that of the dark-grown sample at the same time point. Generally, the expressions of *EGL, GL3*, and *TTG1* only slightly fluctuated, but melatonin-treated samples continued to express higher levels of these genes than the control (Figure 9).

### Effect of melatonin on TBARS, ROS generation, proline levels, and antioxidant enzymes activities in cabbage sprouts

**Figure 10:**
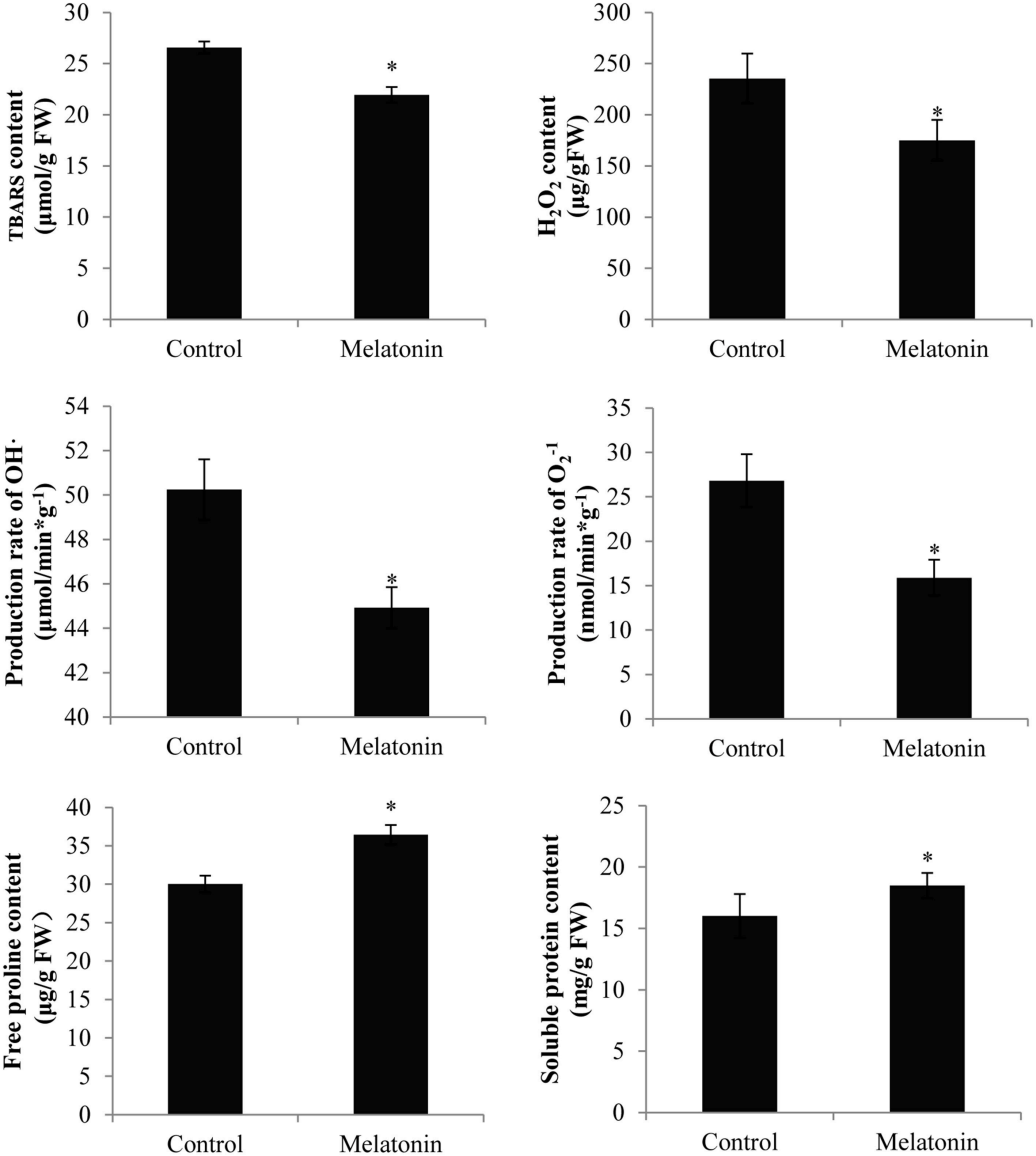
**Melatonin treated cabbage seedlings showed lower radical levels and high proline and soluble protein content**. The concentration of melatonin pretreatment is 1000 μmol /L. Vertical bars at each column represent standard deviation of three replications. ^*^Significant difference between this column and control at *P* < 0.05.

**Figure 11:**
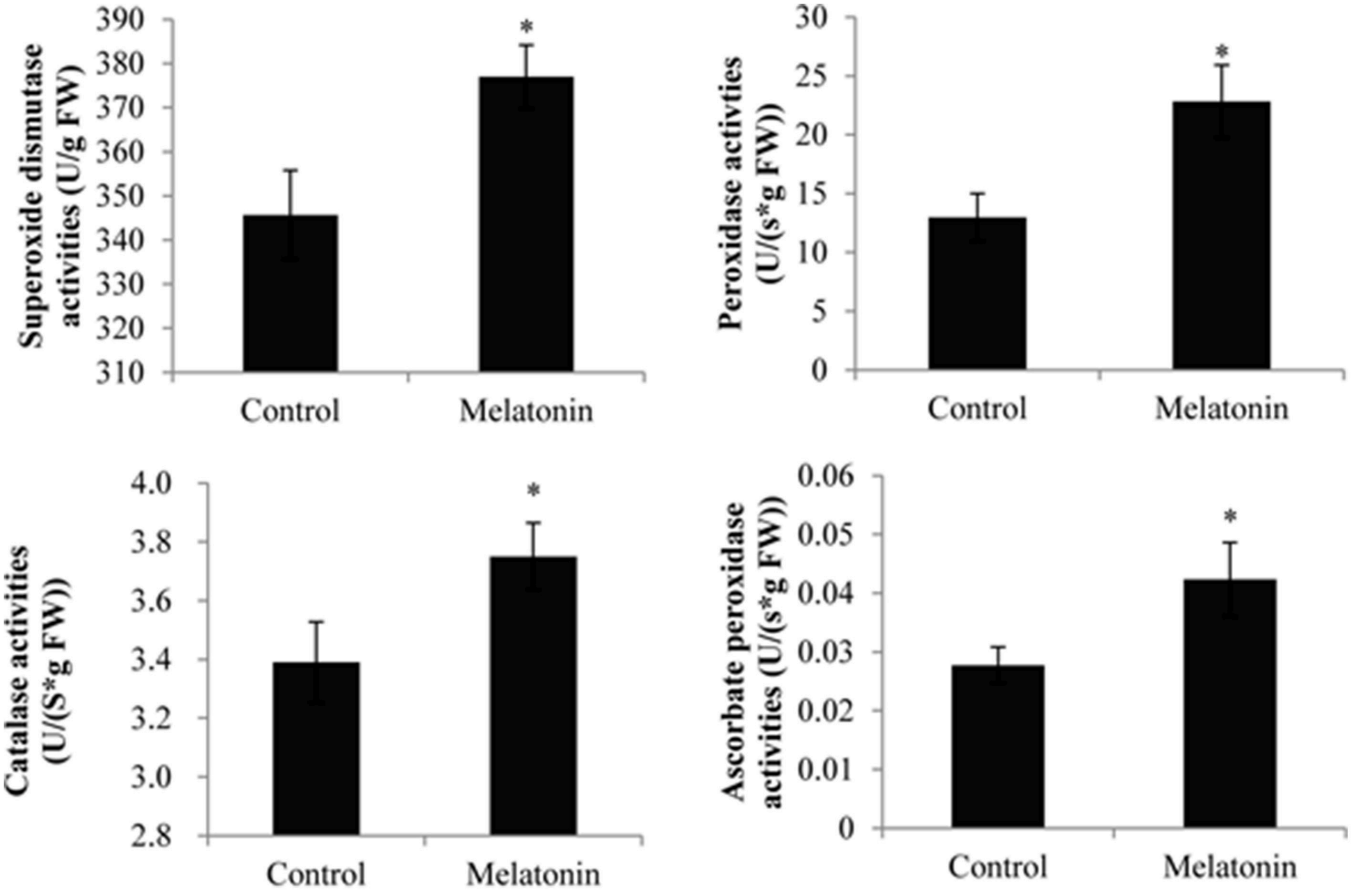
**Melatonin treated cabbage seedlings showed high antioxidant enzyme activities**. The concentration of melatonin pretreatment is 1000 μmol /L. Vertical bars at each column represent standard deviation of three replications. ^*^Significant difference between this column and control at *P* < 0.05.

Melatonin treatment not only promoted anthocyanin accumulation in cabbage but also improved the capacity to resist adverse conditions. Figure 10 shows, that the ROS levels, including the level of H_2_O_2_ and the production rates of OH^·^ and ${\text{O}}_{2}^{-}$, were all decreased in melatonin-treated samples. This decrease was associated with the rapid drop in the TBARS levels in these samples (Figure 10). Adverse environments induce oxidative stress and lipid peroxidation, and changes in the TBARS level have been suggested as indicators of the integrity of cell membranes in plants subjected to stress. Melatonin and anthocyanin can both scavenge excess ROS. Proline overproduction is a well-known response to adverse conditions in plants, and this amino acid is non-toxic, even at high concentrations. Soluble protein has a similar effect. Melatonin treatment increased the endogenous concentration of soluble protein and proline in cabbage compared with the control (Figure 10). These compounds protect plants from stress in various ways, including contributions to cellular osmotic adjustment, the protection of membrane integrity, and the stabilization of enzymes/proteins. Given, that certain agents were observed to alleviate stress and exert beneficial effects, the study of individual antioxidant enzymes was deemed necessary. Melatonin treatment increased the SOD activities by 9%, the CAT activity by 10%, the POD activity by 75%, and the APX activity by 53% (Figure 11). These enzymes are all important antioxidants, that contribute to detoxification of ROS. Melatonin treatment significantly promoted the antioxidant enzymes activities, especially those of peroxidase and ascorbate peroxidase. This finding is consistent with the results of our previous study (Zhang N. et al., 2014).

## Discussion

Anthocyanin biosynthesis is regulated primarily at the transcriptional level. The biosynthesis of anthocyanins is derived from a branch of the flavonoid pathway. PAL and C4H are two key catalytic enzymes upstream of the flavonoid biosynthesis pathway that catalyze phenylalanine to 4-coumaroyl-CoA. They participate in many common metabolic pathways. Therefore, the expression levels of the two structural genes were not as important as those of genes downstream of the synthesis of anthocyanin. CHS facilitates a committed step by condensing one molecule of p-coumaroyl-CoA with three molecules of malonyl-CoA to produce tetrahydroxychalcone. The expression of CHS in white cabbage increased 80 times in response to light compared with dark conditions (Figure 7). A detailed functional analysis of the CHS gene promoter revealed a light-responsive unit carrying a G-box and a MYB recognition element (Hartmann et al., 1998). The G-box can bind bHLH factors. In our result, CHS gene exhibited a relatively strong response to a light signal. This result may be related to the light-responsive unit in the CHS promoter, which may also be responsible for the upregulation of CHS gene expression in melatonin-treated samples. The F3H promoter contains two putative MYB-recognition elements and three ACGT-containing elements, and these motifs are necessary to confer light responsiveness (Zhu et al., 2015). The expression of F3H in white cabbage also strongly responded to light signals, increasing by approximately 50-fold compared with dark conditions (Figure 7). Similarly, other upregulated genes included DFR (23-fold higher), LDOX (14-fold higher), CHI (9-fold higher), GST (9-fold higher), and UFGT (7-fold higher; Figure 7). Interestingly, all highly light-responsive genes were significantly upregulated in white cabbage in response to light but were minimally affected in red cabbage (2.5-fold maximum change; Figures 5–7). In red cabbage, these genes were expressed at high levels in the dark (Figure 5A). We speculated, that the motifs in the promoters of these genes may differ between white cabbage and red cabbage. However, more experimental evidence is needed to confirm this speculation. These genes were upregulated in response to melatonin, irrespective of light treatment. Moreover, anthocyanin accumulation in response to melatonin differed between white and red cabbage (Figures 1, 2). Red cabbage showed a significant response to a very low concentration of melatonin (10 μmol/L), whereas white cabbage responded to higher concentrations of melatonin (Figure 2). However, the anthocyanin content exhibited larger fluctuations, which indicated that red cabbage is more sensitive to melatonin, whereas white cabbage had higher potential in anthocyanin synthesis.

Transcription factors are crucial in the regulation of anthocyanin biosynthesis. Deficiencies in related transcription factors specifically inhibit the expression of anthocyanin biosynthesis genes, but their ectopic expression can activate the entire anthocyanin pathway (Xu et al., 2015). The over-expression of PAP1, which was the first isolated MYB member of this complex in Arabidopsis, results in purple-colored leaves that contain increased levels of anthocyanin and quercetin glycosides (Tohge et al., 2005). MYB and bHLH TFs are found in all eukaryotes and are among the largest families of plant TFs (Feller et al., 2011). We analyzed the expression of related MYB transcription factors, including MYB12, MYB111, PAP1, PAP2, MYB75, and the negative regulator MYBL2, in white cabbage. We found, that melatonin treatment upregulated the positive regulators in most tested samples and downregulated the negative regulator MYBL2 (Figure 8). The bHLH proteins, such as TT8, GL3, and EGL3, participate in anthocyanin biosynthesis and interact with various MYBs. bHLHs contain an N-terminal arginine residue, which is conserved among the bHLH TFs. It can interact with the R3 repeat in MYBs. MYBs and the bHLH proteins, together with TTG1, can form a ternary protein complex named MBW. TTG1 is crucial for the activity of this complex in plants because it regulates both the specific activity (i.e., interactions with other proteins or DNA) and the quantity (e.g., stability and localization) of the MBW complex (Baudry et al., 2004). In our study, melatonin treatment upregulated the expression of MBW-related transcription factors (Figure 8). Many reports have concluded, that melatonin affects various biological processes by regulating the expressions of many transcription factors. For example, melatonin treatment upregulated the expression of class A1 heat-shock factors and consequently improved the thermo-tolerance of Arabidopsis (Shi et al., 2015b). Melatonin also alleviated cold stress by mediating several cold-related genes, including C-REPEAT-BINDING FACTORs (CBFs)/Drought Response Element Binding factors (DREBs), COR15a, and three transcription factors [CAMTA1, ZINC FINGER OF ARABIDOPSIS THALIANA 10 (ZAT10), and ZAT12; (Bajwa et al., 2014; Shi and Chan, 2014)].

Many signaling molecules can affect anthocyanin synthesis. Hormones can also affect the synthesis of anthocyanin. ABA is involved in the regulation of ripening related to anthocyanin biosynthesis in non-climacteric fruits. Silencing an ABA pathway gene, *FaNCED1*, resulted in decreased levels of ABA, and non-colored strawberry fruits. Furthermore, the silencing of a putative ABA receptor also inhibited the ripening and anthocyanin accumulation of strawberries (Jia et al., 2011). Ethylene inhibited sugar- and photosynthesis-induced anthocyanin accumulation in *Arabidopsis* by suppressing the expression of the MBW complex. Ethylene can also inhibit anthocyanin accumulation induced by sucrose and light by suppressing the expression of *TT8, GL3*, and *PAP1* while concomitantly stimulating the expression of the negative regulator *MYBL2* (Jeong et al., 2010). Cytokinins have been found to enhance light- and sugar-induced anthocyanin biosynthesis in *Arabidopsis* (Das et al., 2012). The endogenous application of auxin has been found to retard anthocyanin accumulation in grapevine (Jeong et al., 2004; Wheeler et al., 2009). Moreover, cell culture studies showed, that different auxins regulate anthocyanin biosynthesis, mainly by inducing the expression of *TT8, GL3*, and *PAP1* (Liu et al., 2014). Jasmonates have been found to affect color formation in apples and grapevine, possibly by interacting with ethylene biosynthesis (Fan et al., 1998; Rudell et al., 2005). BRs negatively regulate JA-induced anthocyanin accumulation by inhibiting MBW complexes (Peng et al., 2011). Furthermore, gibberellins can retard the ripening-related accumulation of anthocyanin in fruits (Awad and De Jager, 2002). The DELLA and JASMONATE-ZIM DOMAIN (JAZ) proteins act as key crosstalk components of the GA and JA signaling cascades, respectively, and interact with different partners of the MBW complex (Qi et al., 2014). Sucrose can also affect anthocyanin accumulation. Specifically, it synergistically interacts with phosphate and ABA and with JA, cytokinins, and other hormones involved in the responses to various biotic and abiotic stresses. Moreover, it antagonistically interacts with GA, ethylene, and BRs (Li et al., 2014; Shi and Xie, 2014). In our study, the expression levels of almost all genes, that participate in anthocyanin biosynthesis and regulation fluctuated in response to melatonin treatment (Figures 6–9). However, melatonin did not target one gene or a group of genes to regulate anthocyanin accumulation; instead, melatonin exerted a comprehensive effect.

Environmental factors can also affect the synthesis of anthocyanin. Light exposure can increase the concentration of anthocyanins. In our study, a light signal was sufficient to elicit a large increase in the transcription of the transcriptional activation complex and consequently, the biosynthetic pathway. In addition to light intensity, the light quality also affects the biosynthesis of anthocyanins, especially UV light. However, other specific light qualities (e.g., blue light) have also been associated with the regulation of anthocyanin biosynthesis (Ubi et al., 2006; Li et al., 2013). Low temperatures are well known to induce anthocyanin biosynthesis in plants, especially in light conditions (Catalá et al., 2011). Excess nitrogen application has been found to decrease the anthocyanin content and the contents of other phenolic compounds (Stefanelli et al., 2010). In Arabidopsis, higher nitrogen concentrations reportedly decrease in the expression levels of PAP1 and TT8, which are part of the TTG1–GL3/TT8–PAP1 (WD40–bHLH–MYB) anthocyanin biosynthesis regulatory complex, and increase in the expression levels of three lateral organ-boundary domain genes (LBD37, LBD38, and LBD39), which act as negative regulators of anthocyanin biosynthesis (Zhou et al., 2012). Nitrogen deficiency (low N/C balance) also triggers anthocyanin accumulation in seedlings and rosette leaves, especially at low temperatures, by inducing PAP1, PAP2, and GL3 expression (Lillo et al., 2008; Nemie-Feyissa et al., 2014; Shi and Xie, 2014).

Hu et al. found, that black corn exhibits higher antioxidant activity than yellow and white corn varieties (Hu and Xu, 2011). Moreover, Harakotr et al. found, that the purplish black genotype (KKU-WX111031) consistently exhibited the highest levels of anthocyanins and antioxidant activity among the tested genotypes (Harakotr et al., 2014). Studies of purple potatoes identified that anthocyanins significantly contribute to antioxidant activity (Hu et al., 2016). The health benefits of anthocyanins in purple vegetables have been attributed to their high antioxidant activities.

Anthocyanins appear to protect against both abiotic and biotic stressors. They may reduce the propensity for photoinhibition or mitigate the effects of drought, salinity, heavy metal, or oxidative stress (Chalker-Scott, 1999; Gould, 2004; Agati and Tattini, 2010; Falcone Ferreyra et al., 2012). Different environmental stressors can lead to the generation of ROS in various cellular compartments, e.g., mitochondria and chloroplasts. Melatonin and anthocyanin both exhibit antioxidant capacity. Specifically, melatonin treatment not only upregulated the levels of anthocyanin but also downregulated the ROS levels (Figures 2, 10). Moreover, melatonin treatment increased the activities of antioxidant enzymes (Figure 11), which resulted in a higher ROS-scavenging potential. Proline and soluble protein play important roles in cabbage plants exposed to osmotic stress. These osmoregulation substances were also upregulated in our study (Figure 10). These changes may all improve the ability of cabbage seedlings to resist adverse conditions.

Melatonin has been proven to increase lycopene levels during the post-harvest life of tomato fruits (Sun et al., 2015). Specifically, the expression levels of the SlPSY1 and SlCRTISO genes, which are involved in lycopene biosynthesis, were upregulated in melatonin-treated tomatoes. We also detected the anthocyanin content in melatonin-treated samples (data not shown). We found, that melatonin not only regulated the color of tomatoes by affecting lycopene levels but also affected the anthocyanin content. This phenomenon observed in tomatoes is consistent with, that observed in cabbage. We previously found plant materials can absorb that melatonin. Melatonin pretreatment can significantly increase the levels of melatonin in treated samples, such as tomato fruits and cucumber seeds, whereas the levels of melatonin decreased during ripening and germination (Zhang H. J. et al., 2014; Sun et al., 2015). In this experiment, we also measured the melatonin levels in white cabbage seeds after pre-sowing with melatonin (1000 μmol/L) and seedlings cultured for 1 week after germination (Figure S1). We found, that cabbage seeds absorbed large amounts of melatonin from the soaking solutions. As the plants grew, the melatonin levels decreased, which indicated, that melatonin participates in growth. This finding is consistent with the results of our previous work. Exogenous melatonin application is an effective method to improve the melatonin levels and adversity resistance of plants. Specifically, exogenous melatonin applied to Bermuda grass conferred improved salt, drought, and cold stress resistances (Shi et al., 2015a). Arabidopsis grown in the melatonin-supplemented culture media showed higher survival rates in response to heat shock (Shi et al., 2015b), and applying the optimal dose of melatonin effectively ameliorated Cd-induced phytotoxicity in tomato by affecting Cd transport (Harson et al., 2015). Moreover, melatonin has been proved to scavenge ROS and improve antioxidant activities (Zhang et al., 2013; Zhang H. J. et al., 2014; Li et al., 2015; Shi et al., 2015c), which also corroborates our results.

## Conclusion

Melatonin treatment prior to sowing resulted in a darker red color in cabbage seedlings, and this color is due to anthocyanin accumulation. Melatonin treatment improved the anthocyanin accumulation in white and red cabbage. The expression levels of anthocyanin biosynthesis pathway genes, i.e., PAL, C4H, CHS, CHI, F3H, F3′H, DFR, LDOX, UFGT, and GST, were upregulated in melatonin-treated samples, especially in the presence of light. The upregulated expression of structural genes coincided with a coordinated increase in the transcript levels of MYB transcription factors, bHLH transcription factors, and a WD40 gene. Moreover, the amount of total anthocyanins in cabbage was found to be positively correlated with the total antioxidant power and the content of some osmoregulation substances, suggesting, that anthocyanins may improve the stress resistance of cabbage during growth.

## Author contributions

NZ, QS, and YG designed research; NZ, QS, HL, XL, YC, and HZ performed the experiments; NZ, SL, LZ, YQ, and SR analyzed the data; BZ and YG revised the manuscript.

### Conflict of interest statement

The authors declare that the research was conducted in the absence of any commercial or financial relationships that could be construed as a potential conflict of interest.

## Supplementary material

Table S1 **The genes and primers used for Quantitative PCR**.

Figure S1 **Identification of melatonin by UPLC- ESIMS/MS. (A)** UPLC spectrum of melatonin standard. **(B)** Melatonin levels of cabbage samples presoaked with melatonin solutions (1000 μmol /L) and water control. (1DAT: 1 day after presoaked with melatonin; 7DAT: 7-day-old seedlings). Vertical bars at each column represent standard deviation of three replications.
