# Supplementary material for: Melatonin Improved Anthocyanin Accumulation by Regulating Gene Expressions and Resulted in High Reactive Oxygen Species Scavenging Capacity in Cabbage
Source: Front Plant Sci. 2016 Mar 23;7:197. doi: 10.3389/fpls.2016.00197 (PMC4804130; doi:10.3389/fpls.2016.00197)
Supplement: Table S1 — The genes and primers used for Quantitative PCR. [file Table1.DOC]

| **Gene name** | **Accession** | **Sequence** |
| --- | --- | --- |
| Boactin | AF044573 | CTGTGACAATGGTACCGGAATG |
|  |  | ACAGCCCTGGGAGCATCA |
| BoPAL | BH716217 | GAACCAAGAACGGTGTCGC |
|  |  | GAGAGGGAGGGAAGGAGTGAT |
| BoC4H | XM_013768181 | GCCGTTCCTTAGAGGCTACTTG |
|  |  | CCTTGTTGATTTCTCCCTTCTGTT |
| BoCHS | EF408921 | GCGCATGTGCGACAAGTCGAC |
|  |  | CCTGTCGAGCGTCGAGAGAAGGA |
| BoCHI | EU402417 | TCAAGTTGATTCCGTTACTTTTCCA |
|  |  | ATGACGGTGAAGATCACAAACTTTC |
| BoF3H | DQ288239 | AATGAGAAAAGCCCAAGAAGAGC |
|  |  | TGGTGGATGGAGCCTGAAAT |
| BoF3'H | BH675335 | TTCCGTACCTTCAGGCGGTTATCAA |
|  |  | CTTTGGGGATATGATAGCCGTTGATC |
| BoDFR | AY228487 | GCTCTCTCCTATCACTCGTAACGA |
|  |  | GTCGCATCGTGAGAGGAACAAA |
| BoLDOX | AY228485 | GTGGACAGCTTGAGTGGGAAGATTAC |
|  |  | GTACTCACTCGTAGCTTCAATGTAATCAG |
| BoUFGT | XM_013765032 | GTGAATGATGGGTGTATGGAGG |
|  |  | TCGGCACGAGGTAAAGCAA |
| BoGST | BH738469 | CTTGTAGCCATTTGGTCAA |
|  |  | GAGACTTGCCCAAAAGGTTCGT |
| BoMYB12.1 | Bol001533 | CGAGTGTTGGTGATGGGGAGT |
|  |  | CGACCGACAAGGCTGAAGA |
| BoMYB12.2 | Bol029626 | TTCATCATCAGAAAGCCATCCA |
|  |  | CCTCCTTCGTGACAGGTTACATAG |
| BoMYB111.1 | Bol016599 | CACAATGTCTCCCACAACCTAAA |
|  |  | CTCTATCTCACCATCCAACCACTC |
| BoMYB111.3 | Bol032351 | TGGGTTGGTCTATGTGATTCTTT |
|  |  | ATCCACTTGCGGTATTTCTCC |
| BoMYB75 | Bol042409 | GATAAGTATGGAGAAGGCAAATGG |
|  |  | ATGTTTCTTGCTCAGATGGGTG |
| BoMYBL2 | Bol016164 | CGAACCGACAACGAAGTAAGG |
|  |  | GGGAGCAAACTGGCAAAGAA |
| BoPAP1 | Bol012528 | GCCTTGGACTCAACGACACTAAT |
|  |  | AGCAAACTTTCCCACCACCTAT |
| BoPAP2 | Bol012531 | TTCTTCTACTACACCAGCCCAAAA |
|  |  | CCAAGGCATAGGGGAACAAAT |
| BoTT8 | BH450920 | CCAATAGTTTAGATACACACATGGACATG |
|  |  | TCTTTGACATTCTCAACTCTCCACGATAT |
| BoTTG1 | BH653524 | AGTTGCAGTGGTCGGCTTCTC |
|  |  | AATACGAACCTCAAACTCTAAGGAGCT |
| BoEGL3 | EX078387 | AACTGTCAATTGCAAGCATAAAGGGACA |
|  |  | TGTTTGAATCACTGAGTTCATAAGATTGGA |
| BoGL3 | Bol014556 | AACTGCGATAAGCGGTCAAG |
|  |  | GGTTTCATCCCCAGCCACT |

The accession number is from GenBank <http://www.ncbi.nlm.nih.gov/genbank/> and Bolbase <http://www.ocri-genomics.org/bolbase> which is a database of Brassica Oleracea.
