# Supplementary figures and images for: Melatonin Improved Anthocyanin Accumulation by Regulating Gene Expressions and Resulted in High Reactive Oxygen Species Scavenging Capacity in Cabbage
Source: Front Plant Sci. 2016 Mar 23;7:197. doi: 10.3389/fpls.2016.00197 (PMC4804130; doi:10.3389/fpls.2016.00197)

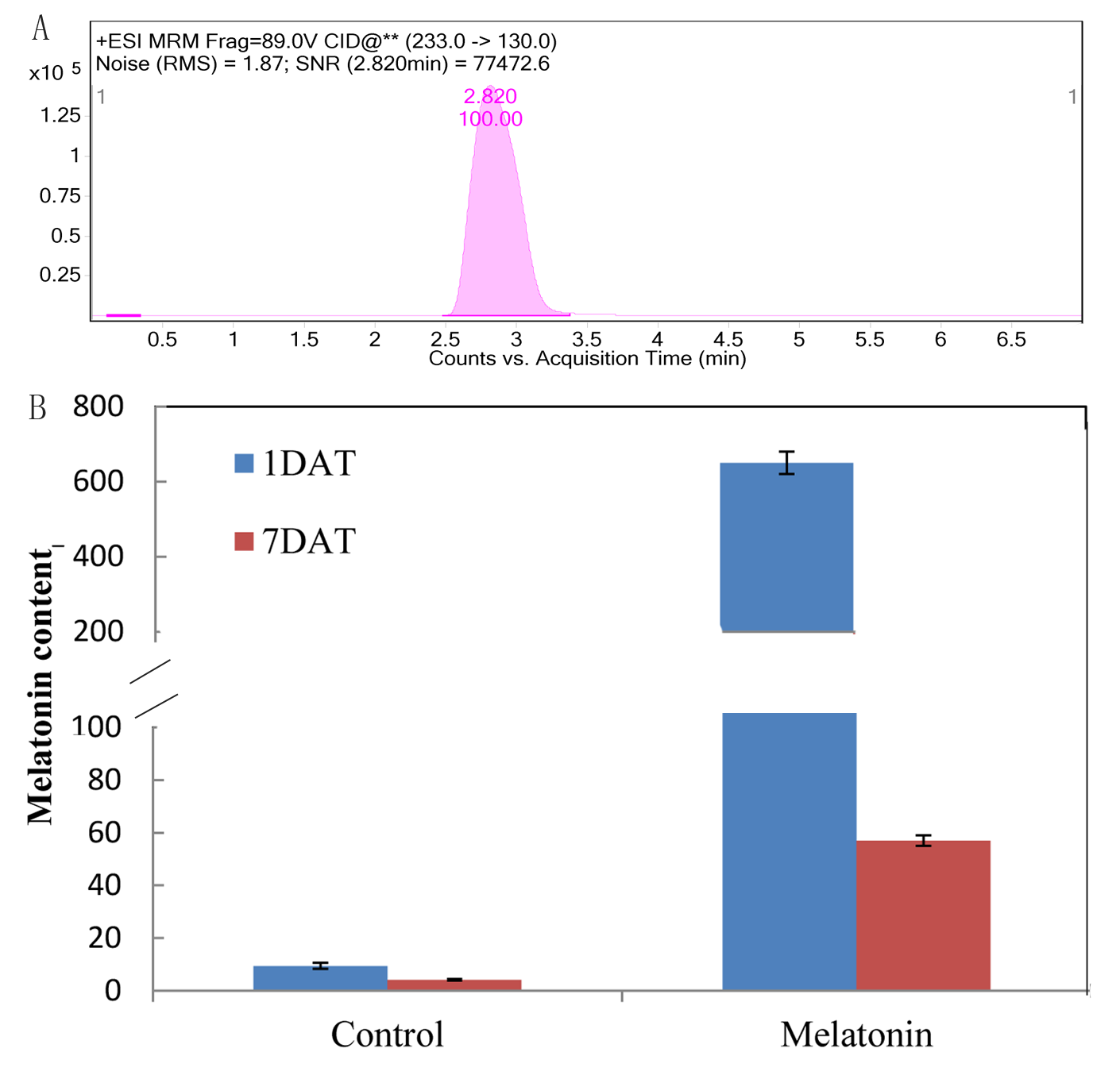

Supplement: Figure S1 — Identification of melatonin by UPLC- ESIMS/MS. (A) UPLC spectrum of melatonin standard. (B) Melatonin levels of cabbage samples presoaked with melatonin solutions (1000 μmol /L) and water control. (1DAT: 1 day after presoaked with melatonin; 7DAT: 7-day-old seedlings). Vertical bars at each column represent standard deviation of three replications. [file Image1.TIF]
